# Fluorescence-Based Nanoparticle Tracking Analysis and Flow Cytometry for Characterization of Endothelial Extracellular Vesicle Release

**DOI:** 10.3390/ijms21239278

**Published:** 2020-12-04

**Authors:** Johannes Oesterreicher, Marianne Pultar, Jaana Schneider, Severin Mühleder, Johannes Zipperle, Johannes Grillari, Wolfgang Holnthoner

**Affiliations:** 1 Ludwig Boltzmann Institute for Experimental and Clinical Traumatology, AUVA Research Centre, 1200 Vienna, Austria; johannes.oesterreicher@trauma.lbg.ac.at (J.O.); marianne.pultar@trauma.lbg.ac.at (M.P.); jaana.schneider@trauma.lbg.ac.at (J.S.); johannes.zipperle@trauma.lbg.ac.at (J.Z.); johannes.grillari@trauma.lbg.ac.at (J.G.); 2 Austrian Cluster for Tissue Regeneration, 1200 Vienna, Austria; 3 Molecular Genetics of Angiogenesis Group, Centro Nacional de Investigaciones Cardiovasculares (CNIC), 28029 Madrid, Spain; severin.muehleder@outlook.com; 4 Christian Doppler Laboratory on Biotechnology of Skin Aging, Institute of Molecular Biotechnology, Department of Biotechnology, University of Natural Resources and Life Sciences, 1090 Vienna, Austria

**Keywords:** extracellular vesicles, endothelial cells, nano particle tracking, fluorescence triggering flow cytometry

## Abstract

As extracellular vesicles (EVs) have become a prominent topic in life sciences, a growing number of studies are published on a regular basis addressing their biological relevance and possible applications. Nevertheless, the fundamental question of the true vesicular nature as well as possible influences on the EV secretion behavior have often been not adequately addressed. Furthermore, research regarding endothelial cell-derived EVs (EndoEVs) often focused on the large vesicular fractions comprising of microvesicles (MV) and apoptotic bodies. In this study we aimed to further extend the current knowledge of the influence of pre-isolation conditions, such as cell density and conditioning time, on EndoEV release from human umbilical vein endothelial cells (HUVECs). We combined fluorescence nanoparticle tracking analysis (NTA) and the established fluorescence-triggered flow cytometry (FT-FC) protocol to allow vesicle-specific detection and characterization of size and surface markers. We found significant effects of cell density and conditioning time on both abundance and size distribution of EndoEVs. Additionally, we present detailed information regarding the surface marker display on EVs from different fractions and size ranges. Our data provide crucial relevance for future projects aiming to elucidate EV secretion behavior of endothelial cells. Moreover, we show that the influence of different conditioning parameters on the nature of EndoEVs has to be considered.

## 1. Introduction

Since the advent of EVs as an emerging topic in biomedical research, a myriad of studies suggests their possible application in tissue engineering, regenerative medicine, targeted therapy, as well as cell free alternatives to stem cell-based approaches and as means for diagnostic purposes [1,2,3,4]. These cell-derived particles mediate various biological responses in the organism upon uptake by their target tissue due to the transport of bioactive cargo such as nucleic acids and proteins. Recent evidence highlights their active role in functions such as sustaining homeostasis in various tissues like heart, cartilage and bone, as well as important response pathways resolving in coagulation, inflammation and sepsis [5,6,7,8,9,10]. In addition, EVs from various cellular sources (e.g., mesenchymal stromal cells, tumor cells) have been shown to enhance vascularization by mediating vessel formation and sprouting in different model systems, which makes them a promising tool for tissue engineering in the future [11,12,13]. Based on their specific cargo molecules, which are dependent on cell type and tissue conditions, EVs may also be used as potent biomarkers in the form of liquid biopsies [14,15,16,17,18]. Especially EndoEVs are promising as they are constitutively released into the blood in different quantities and molecular composition following physiological and pathological conditions [4]. Nevertheless, data on EndoEVs are mainly limited to the larger fractions comprising MVs and apoptotic bodies in regards to their biological relevance in different experimental settings [19,20,21]. Studies focusing on small EndoEVs showing their role in cell signaling, immunomodulation and myocardial regeneration still limit the detection of EVs to “scatter mode” of NTA, transmission electron microscopy, Western blot and enzyme-linked immunosorbent assay (ELISA) for EV characterization [9,22,23]. These generally sophisticated methods, except transmission electron microscopy, exhibit difficulties in distinguishment of EVs and other nano-sized contaminants which may have great impact both in a quantitative and a qualitative manner. Furthermore, the role of cell confluence or cell conditioning times on EV composition have so far not been addressed satisfactorily. In fact, only one study briefly describes the effect of cell confluence on EV secretion from HUVEC, while the EV surface marker profile or the effects of medium conditioning time remain unaddressed [24]. Therefore, in accordance with standardization as proposed in the position statement paper “Minimal Information for Studies of Extracellular Vesicles 2018” (MISEV) by the International Society for Extracellular Vesicles (ISEV) in 2018, we aimed to extend current knowledge regarding EndoEV characteristics, as well as possible changes in EV secretion of HUVEC using different conditioning strategies. We investigated these changes using fluorescence-based methods to distinguish vesicular bodies from conglomerated proteins and other contaminates [25]. We show that both cell confluence and medium conditioning time have significant influences on total EV secretion as well as on their size distributions, which have to be considered as important experimental variables for future studies of EndoEVs. Additionally, we show the expression levels of CD31, VEGFR2, CD9, CD63 and CD81 on the surface of HUVEC-derived EVs.

## 2. Results

### 2.1. Estimation and Analysis of Endothelial Cell Density and Cell Cycle Distributions

**Figure 1:**
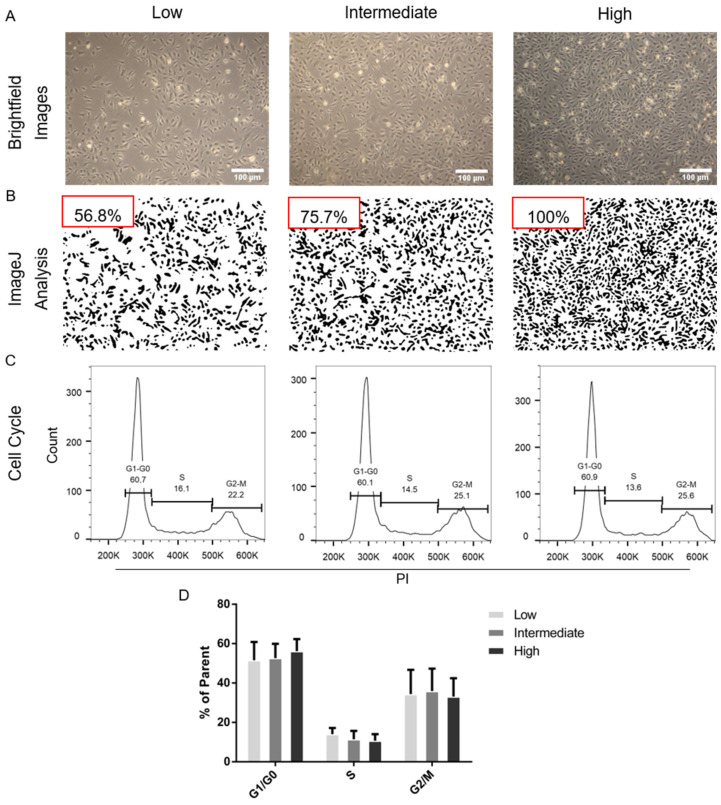
Achieved cell confluences and corresponding cell cycle distributions. Cell numbers for seeding were calculated to reach the respective confluences of 50–60% (Low), 70–80% (Medium) and 90–100% (High) after 24 h growth in endothelial growth medium (EGM-2). (**A**) Brightfield images of cell monolayers were obtained with 4× magnification. White scale bars indicating 100 µm. (**B**) Acquired images were analyzed using ImageJ to gain total surface coverage values for “Low” (56.8%) and “Medium” (75.7%) in relation to “High” (set as 100%). (**C**) Representative histograms plotted against propidium iodide fluorescence intensity of different confluence groups. (**D**) Quantification of the cell cycle distribution. “Low” shows mean values of 51.6% of the parent population in G1–G0, 14.0% in S and 34.4% in G2-M. 52.7% 56.2% in G1–G0, 11.5% and 10.7% in S and 35.9% and 33.1% in G2-M were detected for the “Intermediate” and “High” confluence groups, respectively. EGM-2 figure endothelial growth medium; (**A**–**C**) representative samples, (**D**) *n* = 2 cell donors.

For validation of the influence of cell layer densities on HUVEC different cell numbers were seeded to achieve respective confluence levels of “Low”, “Medium” and “High”. Brightfield imaging (Figure 1A) suggests that intended confluency levels were achieved. The additionally performed confluence analysis with ImageJ resulted in 56.8% for “Low” and 75.7% for “Intermediate” of surface coverage compared to “High”, as shown in Figure 1B. The cell cycle state of the respective confluence groups was assessed via flow cytometric measurement of propidium iodide stained and fixed cells (Figure 1C). “Low” shows the highest percentage of cells in the S phase with a mean of 14.0% compared to “Medium” (11.5%) and “High” (10.7%), suggesting higher proliferative activity. Quantification of cells in G1–G0 and G2–M results in mean values of 51.5%, 52.7%, 56.2% and 34.4%, 35.9% and 33.1% for “Low”, “Medium” and “High”, respectively (Figure 1D).

### 2.2. Establishment of Fluorescence-Triggered Flow Cytometry

**Figure 2:**
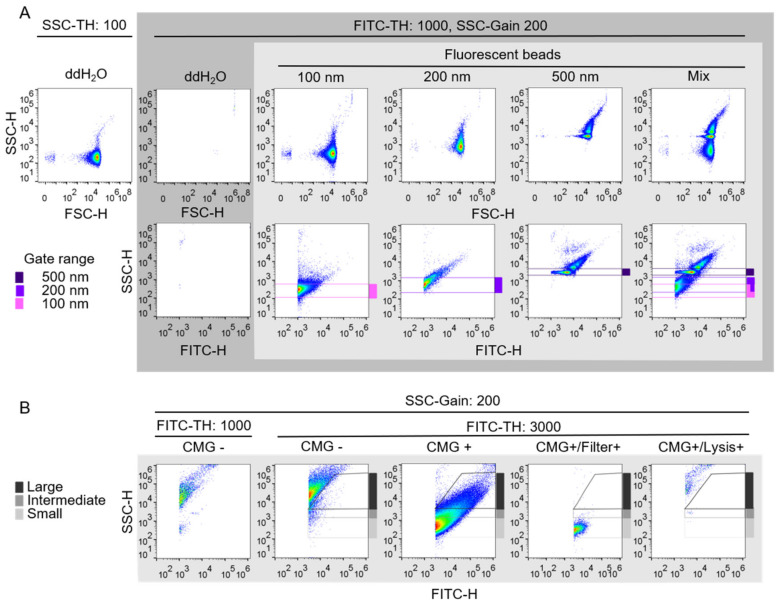
Establishment of fluorescence triggered flow cytometry. (**A**) To exclude unspecific events of particles present in commonly used reagents (top left), the size-dependent SSC default event trigger was changed to a FITC-dependent threshold trigger (top, grey box), resulting in a clearance of background events. FITC-labeled silica beads with different sizes (100, 200 and 500 nm) were applied individually and, subsequently, as a mix to initially confirm differentiated detection of nanometer-sized objects (light grey box). To confirm size-dependent FITC intensity, the FSC was changed to FITC-H (bottom, grey box). The SSC gain was increased to enable better separation and identification of the applied beads. Subsequently, initial gating was performed based on the known sizes of the applied beads shown in blue, purple, and pink (bottom, light grey box). (**B**) To distinguish between labeled vesicles and autofluorescence debris, unstained human umbilical vein endothelial cells (HUVECs) S0.5 cell supernatant fraction was analyzed (first from the left) and the FITC threshold was increased to minimize the detection of unspecific events (second from the left). The before applied size gates shown in A were merged to gain size range gates for small (≤200 nm), intermediate (>200–<500 nm) and large EVs (≥500 nm) before application of CellMask Green-stained HUVECs S0.5 for conformation of enabled detection (third from the left). Filtration of the S0.5 sample with a 0.22 µm polyvinylidene fluoride syringe filter results in loss of signals above the ≤200 nm gate, indicating correct size approximation (fourth from the left). Loss of signal in extracellular vesicle gates after lysis as proof of the vesicular nature of detected events (fifth from the left).

Fluorescence-triggered flow cytometry (FT-FC) was implemented in order to detect single extracellular vesicles and to approximate their size as well as the presence of surface markers. Since particles in the nanometer size range do not produce a forward scatter signal comparable to those of cells according to the Mie scatter theory, the FSC-based detection was changed to a fluorescent intensity-based detection level (Figure 2A) [26]. Further, to exclude any detection of unspecific events and to reduce the background, the threshold for a minimum fluorescent intensity in the FITC channel was set to 1000. This adaption reduced the background signal measured in water to a minimum of detected events. (Figure 2A, dark grey box). The lipid membrane dye cell mask green (CMG) was used to produce green fluorescently labeled extracellular vesicles, which allowed the detection in the FITC-triggered protocol as well as a distinction between extracellular particles and vesicles. Fluorescently labeled silica beads, which produce a comparable emission spectrum to the stained vesicles, were used in different sizes in order to approximate size range gates (Figure 2A light grey box) [27]. The SSC based size gates set according to the 100, 200 and 500 nm FITC-labeled silica beads where merged to a small (≤200 nm), intermediate (>200–<500 nm) and large EV (≥500 nm) size range gate (Figure 2B). The background signal of biological samples was reduced by increasing the FITC threshold to 3000 (Figure 2B first and second plots from the left). In order to evaluate the autofluorescence as well as the vesicular nature and integrity of all extracellular vesicles secreted by endothelial cells, the conditioned medium, clarified from cells, cell debris and big conglomerates (S0.5) from HUVEC, was used for the measurements (Figure 2B, first and second plots from the left). The size approximation and the vesicular nature and integrity of the measured biological HUVEC-derived samples were verified by a 0.22 µm filtration step and the lysis of the sample (Figure 2B fourth and fifth plot from the left), which resulted in the loss of signal above 200 nm and in all detection gates, respectively.

### 2.3. Characterization of Enriched EndoEV Fractions

**Figure 3:**
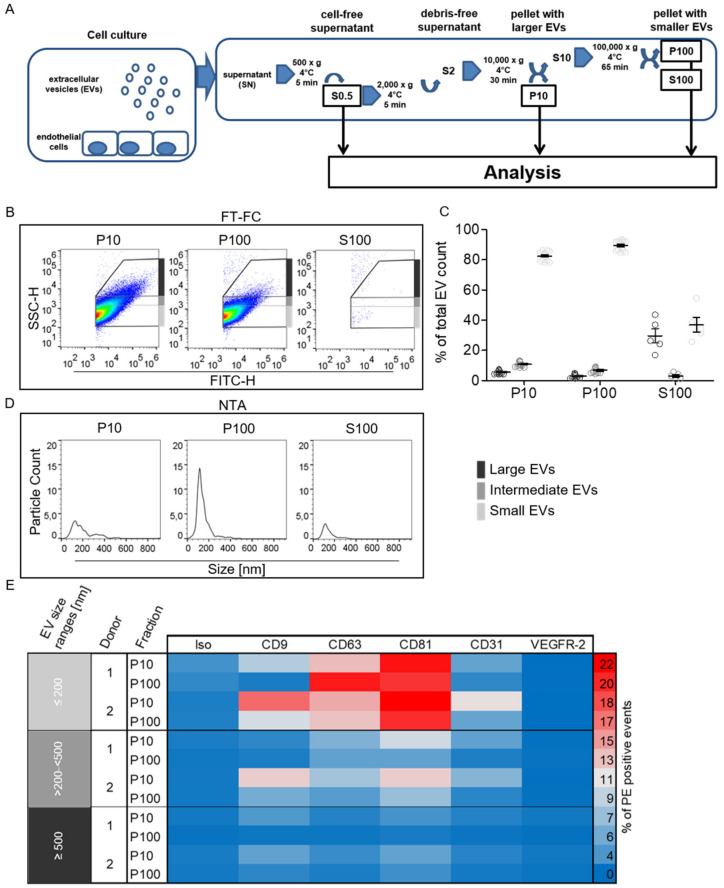
Distinct characteristics of human umbilical vein endothelial cell derived extracellular vesicles EVs. (**A**) The obtained cell culture supernatants (SN) were cleared from debris and large particle conglomerates via centrifugation at 500 and 2000× *g* for 5 min each. The cleared SN (S0.5) was used to analyze total particle and extracellular vesicle (EV) count via scatter and fluorescence mode nanoparticle tracking analysis (NTA). Their total size distribution was assessed via fluorescence-triggered flow cytometry (FT-FC). The large and denser subfraction of EVs was obtained by centrifugation for 30 min at 10,000× *g* (P10). Small and less dense EVs were enriched by ultracentrifugation for 65 min at 100,000× *g* (P100). Both subfractions were analyzed via FT-FC to obtain information on the respective surface marker composition. The remaining supernatant after the differential centrifugation procedure (S100) was analyzed for particle and EV count in order to check the efficiency of the enrichment. (**B**) Resulting representative density scatter plots of FT-FC analyzed EV enrichment fractions show less events detected in large EV gates (dark grey) for P100 enrichment fraction as compared to P10. Only a few events could be detected in S100 indicating a successful enrichment. (**C**) Quantification of EV presence in respective size range gates for FT-FC, showing increased proportion of “Small” vesicles (light grey) with a mean value of 89% and a decrease of “Intermediate” (grey) and “Large” (dark grey) vesicles with a mean of 7% and 3% in P100 compared to P10 with 83%, 11% and 6%, respectively. (**D**) Size distributions and particle counts of enrichment fractions analyzed via scatter mode NTA, for a representative sample. (**E**) Heatmap depicting percentages of CMG-stained and antibody-labeled EVs in the different size ranges (light grey, grey and dark grey), positive for the respective protein target indicated via color code subdivided in donor and individual enrichment fraction. Error bars indicate the mean values ± standard error of mean in scatter plots. (**B**–**D**) Representative samples, (**D**) *n* = 2 cell donors.

In order to determine size distributions and marker protein expression in different EV enrichment fractions, FT-FC and scatter mode NTA measurements were performed. Enrichment fractions were obtained by differential ultracentrifugation of the cell supernatant (Figure 3A). The small vesicular fraction (P100) shows a uniform size distribution when compared to the large vesicular fraction (P10) in both NTA and FT-FC (Figure 3B,C). Quantification of the FT-FC data of the enrichment fractions shows increased small vesicle presence in P100 (with a mean of 90% of total vesicle presence) in comparison to P10 (mean value of 83%) and S100 (mean value of 37%) (Figure 3D). S100 which was used as the control for the enrichment procedure shows overall reduced numbers of events both in NTA and FT-FC. Missing percentages to 100% were detected outside the EV gates. The immunolabeling of the obtained fractions and subsequent analyses via FT-FC yields different levels of investigated surface markers present on the EVs throughout the size ranges. In the heatmap, shown in Figure 3E, the percentage of detected EVs positive for CD9, CD81, CD63 as well as for endothelial cell-specific markers CD31 and vascular endothelial growth factor receptor-2 (VEGFR-2) from the different enrichment fractions are indicated by a colour code. The marker expression profile shows higher presence of EV- specific CD63 and CD81 in P10 and P100 in the small vesicle range (<200 nm), than in the intermediate and large ranges, as well as the existence of the endothelial marker CD31 in P10 on vesicles in the small vesicle range (<200 nm), than in the intermediate and large ranges. VEGFR-2 was not detected in either size range of the different enrichment fractions independent of the cell donor.

### 2.4. Increasing Cell Confluence Influences Release and Size Distribution of EndoEVs

**Figure 4:**
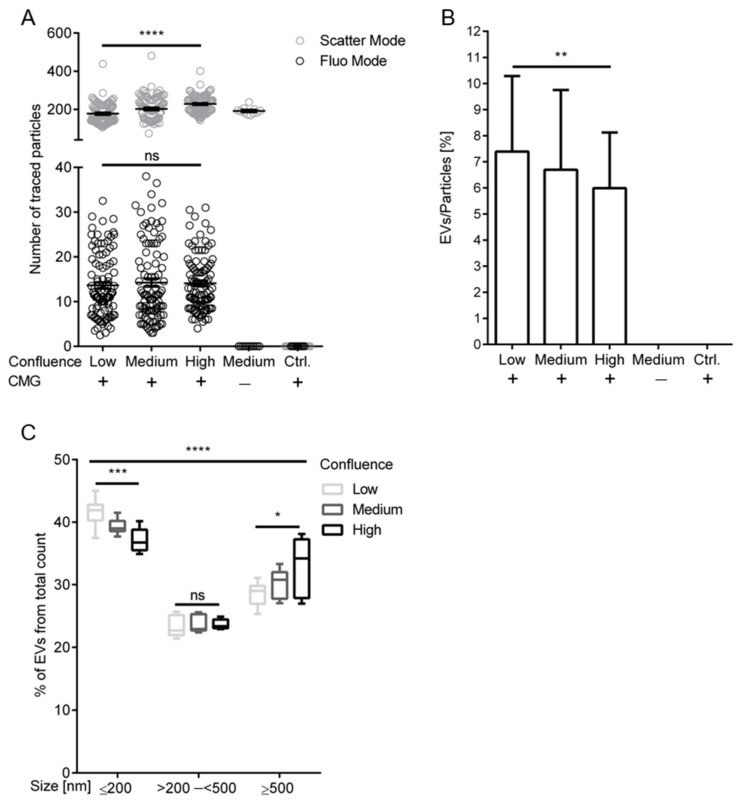
Increasing cell confluence influences release and size of endothelial derived extracellular vesicles. (**A**) Significant differences in traced particles in the scatter mode with 184.9 for “Low”, 228.8 for “Medium” and 236.8 for “High” were detected. No significant difference between particle traces detected in fluorescence mode were found between the mean values of traced particles, 13.6 for “Low”, 14.3 for “Intermediate” and 14.1 for “High”. (**B**) Normalization of fluorescent extracellular vesicles (EVs) against their respective particle numbers detected in the scatter mode revealed increased EV:particle ratios of lower cell density groups with mean values of 7.4% in “Low”, 6.7% in “Medium” and 6.0% in “High”. (**C**) Size distributions of EVs, analyzed via fluorescence-triggered flow cytometry (FT-FC), shows significant shifts from small EV release to increased large vesicle numbers in the different confluence groups. The confluence group “Low” shows an EV size distribution with 41.53% in “Small” (≤200 nm), 23% in “Intermediate” (>200–<500 nm) and 29% in “Large” (≥500 nm). For “Medium” and “High” 39% and 37% in “Small”, 24% and 24% in “Intermediate”, and 30% and 33% in “Large” were detected, respectively. *n* = 3 cell donors, **** = *p* < 0.0001, *** = *p* < 0.001, ** = *p* < 0.01, * = *p* < 0.05, ns = *p* > 0.05. Error bars indicate the mean values ± the standard deviation in bar graphs and ± standard error of mean in scatter plots.

In order to elucidate the possible influence of cell densities on the release of EndoEVs in comparison with non-vesicular particles, the cell and debris clarified conditioned medium containing the whole extracellular vesicle and particle fraction (S0.5) from HUVECs seeded at different densities was investigated via NTA and FT-FC. The analysis of S0.5 by NTA of the cell supernatant derived from different confluences (Low, Medium, High) after 24 h conditioning reveals significant changes on the abundance of total particle numbers, whereas detection of fluorescently labeled EVs shows no significant difference between the groups (Figure 4A). The mean values of the traced particles in the scatter mode which shows significant difference (*p* < 0.001) were 184.9 for “Low”, 228.8 for “Medium” and 236.8 for “High”. No significant difference (*p* > 0.5) was observed between the mean values of traced particles, 13.6 for “Low”, 14.3 for “Medium” and 14.1 for “High” in the fluorescence mode. The normalization of the vesicles detected in the fluorescence mode to their respective total particle traces in the scatter mode reveals a significant decrease in EV:particle ratio following the increase of cell confluence with 7.4% in “Low”, 6.7% in “Medium” and 6% in “High” (Figure 4B). The analyses of the size distributions of the fluorescently labeled EVs by FT-FC revealed differential release of small and large EVs dependent on the cell confluence. The “Low” group shows an EV size distribution with 42% in “Small”, 23% in “Intermediate” and 29% in “Large”. “Medium” and “High” show 39% and 37% in “Small”, 24% and 24% in “Intermediate”, and 31% and 33% in “Large”, respectively. A significant decrease of small (*p* < 0.001) as well as an increase of large (*p* < 0.05) EV abundance was observed in dependence of increasing cell density. No significant difference (*p* > 0.05) in the Intermediate size range of the detected vesicles was seen (Figure 4C).

### 2.5. Increasing Conditioning Time Influences Release and Size Distribution of EndoEVs

**Figure 5:**
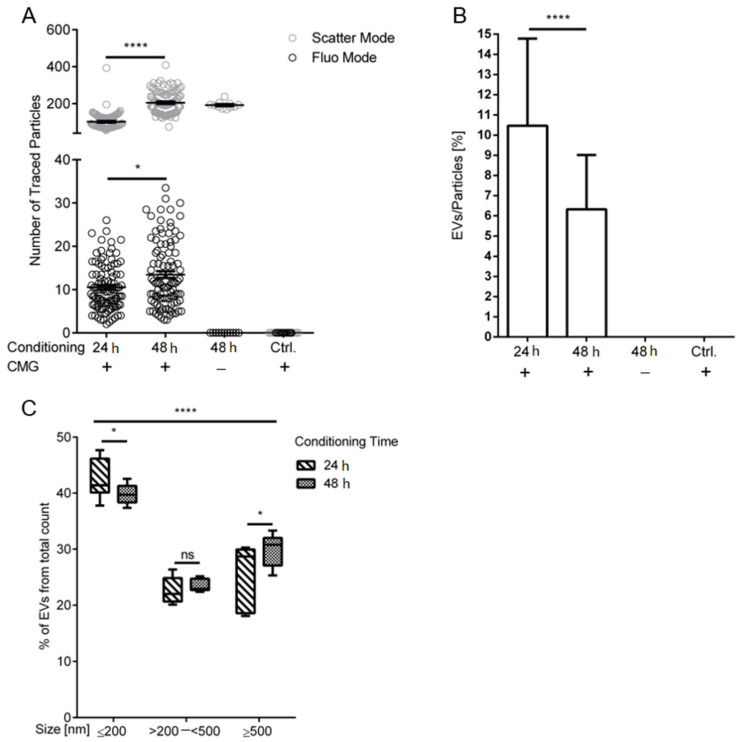
Increasing condition time influences release and size distribution of endothelial derived extracellular vesicles. (**A**) Significant different numbers of traced particles in the scatter mode from different conditioning times were detected with a mean of 102 in the “24 h” group and 205 in the “48 h” group. The number of extracellular vesicles (EVs) detected in the fluorescence mode was significantly different with mean values of 10.6 for the “24 h” group and 13.5 for the “48 h” group. (**B**) Normalization of EVs detected via fluorescence to the total traced particles in the scatter mode shows a significantly reduced EV release over extended conditioning periods, with a mean of 10.5% EVs from the “24 h” group and 6.3% from the “48 h” group. (**C**) Fluorescence-triggered flow cytometry (FT-FC) analyses of EV size distributions reveal an inverse correlation of EV sizes and conditioning times. Mean values of 43% “Small” (≤200 nm), 23% “Intermediate” (>200–<500 nm) and 26% “Large” (≥500 nm) in the respective size gates were detected for the “24 h” group. A percentage of 40% in “Small”, 24% in “Intermediate” and 30% in “Large” were detected in the “48 h” group. *n* = 3 cell donors, **** = *p* < 0.0001, * = *p* < 0.05, ns = *p* > 0.05. Error bars indicate the mean values ± the standard deviation in bar graphs and ± standard error of mean in scatter plots.

In order to reveal possible effects of conditioning periods and the deprivation of growth media supplements on the vesicle release of HUVECs, again the S0.5 fraction from cells at “Medium” confluence, which were conditioned for 24 or 48 h, were analyzed using NTA and FT-FC. NTA analysis shows significant effects of different conditioning times (24 and 48 h) on the total particle (scatter) as well as EV (fluorescence) release of HUVECs seeded at equal densities (Figure 5A). The “24 h” group shows mean values for traced particles of 102 in the scatter mode and 10.55 in the fluorescence mode, whereas the “48 h” group results in 205 and 13.47, respectively, leading to the significant difference between both conditioning times (*p* < 0.0001 for scatter mode and *p* < 0.05 for fluorescence mode). After normalization of the traces detected in fluorescence mode against the traces of particles from the scatter mode, a significant decrease (*p* < 0.0001) of the EV:particle ratio was observed, with 10.46% EVs from the total particle count in the “24 h” group and 6.32% in the “48 h” group. Thereby suggesting a reduction of EV release during extended condition periods (Figure 5B). Furthermore, FT-FC analyses of the size distributions of the total extracellular vesicle fraction results in changes from increased small EV release in “24 h” conditioning to increased large vesicle release in the “48 h” group (Figure 5C). The “24 h” group shows mean values of 43% “Small”, 23% “Intermediate” and 26% “Large”, whereas 40%, 24% and 30% were detected in the “48 h” group, respectively. Missing percentages to 100% were detected outside the EV gates. Significant difference between both conditioning groups in the “Small” and “Large” size range (*p* < 0.05) and no significant difference (*p* > 0.05) in “Intermediate” were seen.

## 3. Discussion

In this study we provide data indicating an influence of cell seeding density and medium conditioning time on the size and number of EVs secreted by HUVEC. Recent work provides evidence for possible influences of cell culture parameters on EV secretion and bioactivity; however, no distinction between EVs or non-vesicular particles was conducted [24]. Therefore, we performed fluorescence mode NTA and FT-FC to allow detection, characterization and specification of lipid layer-enclosed EVs to generate an experimental setup according to proposed MISEV and MIFlowCyt-EV guidelines [25,28]. EVs are getting steadily increasing attention throughout the biomedical research field, with a plethora of publications suggesting a variety of applications for tissue engineering or diagnostic purposes [29,30]. Nevertheless, major obstacles in the endeavor of utilizing the potentials of EVs still remain. Problems such as the uncertainty of the influence of pre-analytical variables like cell culture conditions, or the often unaddressed question of vesicular identity of detected particles, often impede interpretation of results in the field [25,31]. Additionally, despite the well-known importance of endothelial cells in homeostasis and many pathologies, knowledge regarding EndoEVs is still limited and often focused on the investigation of microvesicles [32,33,34,35,36].

Due to the detection limits of conventional flow cytometry, the reported fluorescence trigger protocol was established to circumvent hindering background signals present in commonly used reagents, which allows for specific EV detection and subsequent analysis, as already discussed in great detail by others [37,38,39]. The approximation of microvesicle size is known to be limited due to the low refractive index of 1.39 and a size under the illumination wavelength of 488 nm of lipid nanospheres [40]. The size parameter, which is dependent on the diameter of the particle and the wavelength of the laser, is used to gain information on the underlying scattering principle of nanoparticles in flow cytometry. For EVs in the range of 30–500 nm, the size parameter is 0.19–3.2, which has the consequence that the Mie scattering theory has to be applied, which is dependent on the refraction index as well as on the size parameter of spherical scattering particles [26]. Consequently, the determination of the size was only assessed in form of a size range approximation, since the refractive index of available synthetic standard nanobeads differs from the one of EVs. In our study we used silica beads, which have a refractive index of 1.46, which is closer to the index of EVs when compared to other synthetic nanobeads such as polystyrene beads with a refractive index of 1.6 [27,41]. As described shown in Figure 2, we observed an overlap of 100 and 200 nm fluorescently labeled silica beads, whereas 500 nm beads could be clearly discriminated from the smaller beads. Consequently, the size gates where set as size ranges from small (≤200 nm), intermediate (>200–<500 nm) and large EVs (≥500 nm). Successful verification of the small EV gate was achieved by the measurement of 0.22 µm filtered S0.5 containing only nanoparticles below 220 nm. Despite known pitfalls of EV detection via flow cytometry, our data indicates a successful analysis of surface markers as well as size approximation, which allows for characterization of the different enrichment fractions supporting and complementing the data generated via fluorescence NTA.

The analysis of the debris-free cell supernatant (S0.5) of HUVEC seeded in different cell confluences resulted in distinct EV abundances in the respective samples. The use of this non-enriched fraction allowed analysis of the whole EndoEV population of any size, as further enrichment via ultracentrifugation may influence subsequent acquisition of size and abundance [42]. Our data show significant reduction of the EV:particle ratio with increased cell densities despite the apparent increase in total particle presence. The change in vesicular secretion might be linked to the known influence of cell density leading to the withdrawal from the cell cycle in high confluence state endothelial cells, and would contrast the reported increased small EV secretion in fibroblasts and cancer cell lines [43,44]. Furthermore, cell density has not only been shown to actively modulate proliferation rates but also gene expression and epigenetic regulation of target genes in endothelial cells, which might lead to up and down regulation of EV secretion in a still unknown fashion [45,46,47]. As the total abundance of EVs changed in correlation to the cell density, their size distributions show a shift from higher numbers of small vesicles in lower confluence and increased numbers of large vesicles in the high confluence groups. The observed change in EV size distributions might result from a change in the endosomal biogenesis pathway of EVs, as it has already been shown that lysosomal enzymes tend to accumulate in the quiescent cell state, as well as the influence of confluence on endocytosis in mammalian cells [48]. The mechanism behind the observed changes in the sizes of vesicles, and possibly their biogenesis upon different cell confluence, has to be addressed in future studies by investigation of their respective cargo as well as complementary transcriptional changes in the cells of origin.

The analysis of the vesicular fraction in the context of different conditioning times reveals significant influences on both the EV:particle ratio as well as the size distribution of EVs. The observed change from high EV content to unspecific particles in the analyzed supernatant might as well be caused by the before-mentioned changes in biogenesis pathways. Furthermore, an elongation in the time of serum deprivation might cause an increase in apoptotic body release, which was subsequently detected as the increase in the large EV fraction [49]. Since the nutrient depletion of the media over time might be a cause of the detected changes, this prospect should be investigated in further studies by application of different media volumes, as well as the removal and exchange of the medium. Further research will also have to address the question of influences of EV release per cell in a sophisticated manner. Simple strategies, such as cell counting after conditioning, might not be sufficient as the results may be biased from possible cell death, which, for example, would artificially increase the EV/cell count.

The investigation of the surface markers CD9, CD63, CD81, CD31 and VEGFR-2 of EndoEVs reveals their presence on different subfractions enriched by ultracentrifugation. CD63 and CD81 were found on all different subpopulations regardless of the used cell donor, indicating an EV identity of analyzed particles [50]. The lower presence of CD9 compared to CD63 and CD81 on the analyzed fractions might be considered as an EndoEV characteristic based on similar findings published by Boyer et al., where CD9 could only be detected via mass spectroscopy [51]. As an endothelial-specific marker, CD31 was identified on small EVs, which should be further investigated and confirmed in the future for possible distinctions of EV sources in body fluids in the context of liquid biopsies. Performed studies thereby show the general presence of CD31 on EVs isolated from human serum, as well as its increased abundance in pathologies such as acute coronary syndrome and their specific cargo on diabetes 2, hinting towards their diagnostic potential [52,53]. As CD31 is known to be cytoprotective towards different pro-apoptotic stimuli in endothelial cells, the active secretion via EVs might; therefore, be a possible intercellular response to deprivation of serum during conditioning [49,54]. VEGFR-2 was not detected on any of the investigated fraction and; therefore, might be excluded as a possible cell type-specific marker for EndoEVs originating from HUVEC.

In summary, our data show significant changes in the EV secretion patterns due to different conditioning and cell seeding densities which should be considered for future projects. Additionally, the applied fluorescence NTA and FT-FC were able to show successful differentiation between EVs and non-vesicular particles, which allows for EV specific characterization in contrast to the other widely used methods such as conventional NTA. With the here presented results, we further extend the current knowledge on the influence of cell culture parameters on EndoEV secretion in a vesicle specific manner. Further investigations regarding the pathways which modulate EV release in different endothelial cell types have to consider other vascular beds, in order to reveal organotypic vasculature-specific vesicle releasing mechanisms and understand these cell-derived mediators in health and disease.

## 4. Materials and Methods

### 4.1. Culturing and Conditioning of Cells

The isolation of primary cells was approved by the local ethics committee of the state of Upper Austria with written informed consent by the donors (ethics committee vote #200, 12/05/2005). All data shown were obtained from several independent experiments, where at least two different biological donors were used (indicated by *n* in each figure). HUVECs were isolated according to established protocols, as previously described [55]. The cells were grown in EGM-2 (Lonza, Walkersville, MD, USA) supplemented with 5% fetal calf serum (FCS) at 37 °C and 5% CO_2_ and used in passage 7 for all experiments_._ The cell number used for seeding was calculated on the basis that a confluent layer of HUVECs comprises approximately 4 × 10^4^ cells per cm^2^. Approximately 2 × 10^5^, 1.4 × 10^5^ and 1 × 10^5^ cells were seeded into 6-well plates to reach estimated confluence levels of 50–60%, 70–80% and 90–100% after 24 h in EGM-2, termed as “Low”, “Medium”, and “High”, respectively. For the characterization of EndoEV surface markers, 1.05 × 10^6^ cells were seeded in T75 flasks with EGM-2. Subsequently, the growth medium was removed and the cells were washed three times with PBS 1× (without Ca^++^ Mg^++^) to remove remaining possible contaminants, including EVs from the FCS. A total of 2 and 13 mL (6-well and T75, respectively) of endothelial basal medium (EBM-2, Lonza, Walkersville, MD, USA) were used for conditioning for 24 or 48 h before EV harvesting and storage at 4 °C of the supernatant.

### 4.2. Assessment of Cell Confluences and Cell Cycle Profiles

Beside microscopic evaluation of cell confluence by conventional phase microscopy, images were analyzed for total substrate coverage by cells using ImageJ. To obtain numerical values representing the cell confluence level of each seeding density, the total area covered by cells was set in relation to the highest seeding density. This was performed by first changing the image type to 8-bit before deletion of the background (<30 pixels), followed by contrast enhancement (10%). Subsequently, the signal noise was filtered by applying a bandpass filter and adapting the black-and-white threshold until shape and presence of cell outlines closely resembled the original phase contrast image. Debris and other small objects were removed by applying a median filter adapted to the unspecific objects present in the respective images. The area covered by the cells was automatically selected and the total area given as a numerical value. Furthermore, cell cycle analysis of the different seeding densities was performed via flow cytometry after fixation of the cells in 75% ethanol and subsequent labeling of nucleic acids content by applying a propidium iodide staining solution including RNAse for the removal of cellular RNA (Trisodium citrate *2H_2_O 4 mM, RNAse A 25 µg/mL, Propidium Iodide 50 µg/mL, Triton X-100 0.1% *v*/*v*). After incubation of the cell suspension for 15 min at RT, the cells were analyzed on the flow cytometer Cytoflex (Beckmann Coulter, Carlsbad, CA, USA) at 60 µL/min until 10,000 events in the initial cell gate were achieved. Further analysis including subsequent gating was performed with FlowJow v.10 (Dickinson and Company, Ashland, OR, USA).

### 4.3. Enrichment of EVs by Differential Ultracentrifugation

The clearance and enrichment of EndoEVs from cell culture supernatants was performed by differential ultracentrifugation (Figure 3A). For the general investigation of extracellular particle and vesicle release from HUVECs, the supernatant was only cleared from debris and large conglomerates by centrifugation at 500× *g* for 5 min and subsequent retrieval of the supernatant, herein termed “S0.5”, using the fixed angle rotor (SN867) of the Heraeus Megafuge 16R (Thermo Fisher Scientific, Waltham, MA, USA) centrifuge to avoid the loss of vesicles of any size. Large EVs including microvesicles were enriched by centrifugation of the “S0.5” fraction for a further 5 min at 2000× *g,* before transferring the obtained supernatant “S2” and applying 10,000× *g* for 30 min using the before mentioned fixed angle rotor. The obtained pellet “P10” was resuspended in sterile filtered PBS 1× (without Ca^++^ Mg^++^; 0.22 µm PVDF syringe filter, Roth) and stored at 4 °C until further use. The supernatant (S10) was transferred into ultracentrifugation tubes (Ultra-Clear, Beckmann Coulter Carlsbad, CA, USA) and centrifuged using a swing out rotor setup (SW40.1 Ti) of the Beckman Coulter ultracentrifuge L-100XP (Beckmann Coulter, Carlsbad, CA, USA) at 100,000× *g* for 65 min (including acceleration time) at 4 °C in vacuum. The pellet “P100”, containing the small fraction of EndoEVs, was resuspended in sterile-filtered PBS 1× (without Ca^++^ Mg^++^) and stored at 4 °C, as well as the supernatant “S100”, until further use. Storage time did not exceed 48 h of any sample used. The used centrifugation speeds and times were calculated using the web calculator for specific “cut-off” sizes developed by Livshits et al. [56].

### 4.4. Nanoparticle Tracking Analysis of EndoEV Abundance in Cell Culture Supernatant

To assess the concentration and size distributions of extracellular particles and vesicles derived from the cell debris free supernatant S0.5 of HUVEC, the Zetaview PMX110 device from Particle Metrix (Particle Metrix, Inning a. Ammersee, DEU) was used for nanoparticle tracking analyses. To distinguish non-lipid particles from EVs, the samples were stained with the lipid membrane specific dye CellMask Green (CMG, Invitrogen, UK) and measured using the fluorescence mode of the device. This was performed by addition of a 1:2000 dilution of CMG with sterile filtered PBS 1× (without Ca^++^ Mg^++^) and subsequent incubation for 20 min at 37 °C in the incubator. Thereafter, the samples were further diluted with sterile filtered PBS 1× (without Ca^++^ Mg^++^) to the concentration suggested by the manufacturer for optimal measurements. To prevent bleaching, the laser intensity was reduced to the minimum (shutter 500) for sample application. The measurement was performed using the maximum laser intensity (shutter 32) and a sensitivity setting of 95 in the fluorescence mode. Following the fluorescence measurement, the samples were again measured using the scatter mode with shutter 50 and sensitivity set to 70–80 resulting in the total particle concentration including particles without lipid layers and EVs.

### 4.5. Fluorescence-Triggered Flow Cytometry Analysis of EVs

The established FT-FC protocol is based on the staining of lipid layers using the before mentioned lipid membrane specific dye CMG, and the following detection and triggering of an event in the flow cytometer not based on a scatter signal but on the fluorescence intensity of the detected particle [39]. Thereby, only EVs which are enclosed by a lipid layer which incorporated the dye will be detected excluding the majority of unspecific particles. The protocol additionally allows for size approximations based on the side scatter signal of EVs due to previously performed gating with fluorescently labeled silica beads with defined sizes (FITC labeled silica beads 100, 200 and 500 nm, Kisker Biotech, Steinfurt, DEU). The establishment of the FT-FC protocol including the gating of different size fractions is shown in Figure 2. For the CMG staining and antibody labeling of the respective enrichment fractions, 80 µL of the supernatant or the resuspended EV pellet were transferred to 1.5 mL tubes, before adding 20 µL of the 1:2000 CMG working solution and incubating for 20 min at 37 °C in the dark. Afterwards, 0.5 µL of the respective directly PE-labeled antibodies for CD31 (monoclonal, BD Pharmingen, Clone WM59), CD9, CD63 and CD81 (recombinant, Miltenyi Biotec, respectively Clone REA1071, REA1055, REA513) as well as VEGFR-2 (recombinant, Miltenyi Biotec, Clone REA1046) and the IgG Isotype (BD Pharmingen, Clone, MOPC-21) diluted in 100 µL filtered PBS 1× (without Ca^++^ Mg^++^) were added. The samples were vortexed thoroughly before further incubation on ice in the dark for 30 min. As controls, PBS 1× (without Ca^++^ Mg^++^) with CMG and/or antibodies and EV samples without CMG and/or antibodies were analyzed to ensure no possible antibody conglomerates were detected (data not shown). For the lysis control, 100 µL of RIPA-Lysis buffer (Tris-Cl pH 7.4 50 mM; NaCl 150 mM; Triton X-100 1%; sodium deoxycholate 0.5%; SDS 0.1%; Phenylmethylsulfonyl fluoride 0.1%) was added to the CMG-stained samples and incubated for 30 min on ice before measurement.

### 4.6. Statistical Analysis

The statistical analysis was performed using Graph Pad Prism v.6.01 (GraphPad Software, San Diego, CA, USA). For the comparison of two normally distributed samples, the Student’s *t*-test was conducted. Otherwise the Mann–Whitney test was used. One-Way ANOVA was chosen as the appropriate method to test for significant difference between more than two normally distributed samples, followed by the Tukey’s multiple comparison test. For more than two not-normally distributed groups, the Kruskal–Wallis test and the Tukey’s multiple comparison were performed. Normality was tested via the D’Agostino–Pearson omnibus test. Grouped data was analyzed using the two-way ANOVA. The number of used donors (*n*), the *p*-values and the respective statistical significance are indicated in each figure. The data are plotted as mean with ± standard error of mean in scatter plots and ± standard deviation in bar graphs.

## Abbreviations

EV	Extracellular Vesicle
EndoEV	Endothelial EV
HUVEC	Human Umbilical Vein Endothelial Cell
NTA	Nano Particle Tracking Analysis
FT-FC	Fluorescence Trigger Flow Cytometry
EGM-2	Endothelial Growth Medium
VEGFR-2	Vascular Endothelial Growth Factor Receptor 2
FCS	Fetal Calf Serum
EBM-2	Endothelial Basal Medium
CMG	Cell Mask Green
MV	Microvesicles
MISEV	Minimal Information for Studies of Extracellular Vesicles 2018
ISEV	International Society for Extracellular Vesicles
VEGFR-2	Vascular Endothelial Growth Factor Receptor 2
PI	Propidium Iodide
SDS	Sodium Dodecyl Sulfate

## References

[1] De Jong O.G., van Balkom B.W.M., Schiffelers R.M., Bouten C.V.C., Verhaar M.C. (2014). Extracellular vesicles: Potential roles in regenerative medicine. Front. Immunol..

[2] Tsiapalis D., O’Driscoll L. (2020). Mesenchymal Stem Cell Derived Extracellular Vesicles for Tissue Engineering and Regenerative Medicine Applications. Cells.

[3] Zhou Y., Xiao Y. (2020). The Development of Extracellular Vesicle-Integrated Biomaterials for Bone Regeneration. Adv. Exp. Med. Biol..

[4] Hromada C., Mühleder S., Grillari J., Redl H., Holnthoner W. (2017). Endothelial Extracellular Vesicles-Promises and Challenges. Front. Physiol..

[5] Li Q., Huang Q.-P., Wang Y.-L., Huang Q.-S. (2018). Extracellular vesicle-mediated bone metabolism in the bone microenvironment. J. Bone Miner. Metab..

[6] Hutcheson J.D., Aikawa E. (2018). Extracellular vesicles in cardiovascular homeostasis and disease. Curr. Opin. Cardiol..

[7] Chistiakov D.A., Orekhov A.N., Bobryshev Y.V. (2016). Cardiac Extracellular Vesicles in Normal and Infarcted Heart. Int. J. Mol. Sci..

[8] Holnthoner W., Bonstingl C., Hromada C., Muehleder S., Zipperle J., Stojkovic S., Redl H., Wojta J., Schöchl H., Grillari J. (2017). Endothelial Cell-derived Extracellular Vesicles Size-dependently Exert Procoagulant Activity Detected by Thromboelastometry. Sci. Rep..

[9] Hosseinkhani B., Kuypers S., van den Akker N.M.S., Molin D.G.M., Michiels L. (2018). Extracellular vesicles work as a functional inflammatory mediator between vascular endothelial cells and immune cells. Front. Immunol..

[10] Raeven P., Zipperle J., Drechsler S. (2018). Extracellular Vesicles as Markers and Mediators in Sepsis. Theranostics.

[11] Sheldon H., Heikamp E., Turley H., Dragovic R., Thomas P., Oon C.E., Leek R., Edelmann M., Kessler B., Sainson R.C.A. (2010). New mechanism for Notch signaling to endothelium at a distance by Delta-like 4 incorporation into exosomes. Blood.

[12] Lamichhane T.N., Leung C.A., Douti L.Y., Jay S.M. (2017). Ethanol Induces Enhanced Vascularization Bioactivity of Endothelial Cell-Derived Extracellular Vesicles via Regulation of MicroRNAs and Long Non-Coding RNAs. Sci. Rep..

[13] Gai C., Carpanetto A., Deregibus M.C., Camussi G. (2016). Extracellular vesicle-mediated modulation of angiogenesis. Histol. Histopathol..

[14] Agrahari V., Agrahari V., Burnouf P.-A., Chew C.H., Burnouf T. (2019). Extracellular Microvesicles as New Industrial Therapeutic Frontiers. Trends Biotechnol..

[15] Lener T., Gimona M., Aigner L., Borger V., Buzas E., Camussi G., Chaput N., Chatterjee D., Court F.A., Del Portillo H.A. (2015). Applying extracellular vesicles based therapeutics in clinical trials-an ISEV position paper. J. Extracell. Vesicles.

[16] Muralidharan-Chari V., Clancy J.W., Sedgwick A., D’Souza-Schorey C. (2010). Microvesicles: Mediators of extracellular communication during cancer progression. J. Cell Sci..

[17] Vlassov A.V., Magdaleno S., Setterquist R., Conrad R. (2012). Exosomes: Current knowledge of their composition, biological functions, and diagnostic and therapeutic potentials. Biochim. Biophys. Acta-Gen. Subj..

[18] Barile L., Vassalli G. (2017). Exosomes: Therapy delivery tools and biomarkers of diseases. Pharmacol. Ther..

[19] Paone S., Baxter A.A., Hulett M.D., Poon I.K.H. (2019). Endothelial cell apoptosis and the role of endothelial cell-derived extracellular vesicles in the progression of atherosclerosis. Cell. Mol. Life Sci. CMLS.

[20] Carracedo J., Alique M., Ramírez-Carracedo R., Bodega G., Ramírez R. (2019). Endothelial Extracellular Vesicles Produced by Senescent Cells: Pathophysiological Role in the Cardiovascular Disease Associated with all Types of Diabetes Mellitus. Curr. Vasc. Pharmacol..

[21] Vítková V., Živný J., Janota J. (2018). Endothelial cell-derived microvesicles: Potential mediators and biomarkers of pathologic processes. Biomark. Med..

[22] Crewe C., Joffin N., Rutkowski J.M., Kim M., Zhang F., Towler D.A., Gordillo R., Scherer P.E. (2018). An Endothelial-to-Adipocyte Extracellular Vesicle Axis Governed by Metabolic State. Cell.

[23] Chen C.W., Wang L.L., Zaman S., Gordon J., Arisi M.F., Venkataraman C.M., Chung J.J., Hung G., Gaffey A.C., Spruce L.A. (2018). Sustained release of endothelial progenitor cell-derived extracellular vesicles from shear-thinning hydrogels improves angiogenesis and promotes function after myocardial infarction. Cardiovasc. Res..

[24] Patel D.B., Gray K.M., Santharam Y., Lamichhane T.N., Stroka K.M., Jay S.M. (2017). Impact of cell culture parameters on production and vascularization bioactivity of mesenchymal stem cell-derived extracellular vesicles. Bioeng. Transl. Med..

[25] Théry C., Witwer K.W., Aikawa E., Alcaraz M.J., Anderson J.D., Andriantsitohaina R., Antoniou A., Arab T., Archer F., Atkin-Smith G.K. (2018). Minimal information for studies of extracellular vesicles 2018 (MISEV2018): A position statement of the International Society for Extracellular Vesicles and update of the MISEV2014 guidelines. J. Extracell. Vesicles.

[26] Pu Y., Chen J., Wang W., Alfano R.R., Alfano R.R., Shi L.B.T.-N.B.S. (2019). 11-Basic Optical Scattering Parameter of the Brain and Prostate Tissues in the Spectral Range of 400–2400 nm. Nanophotonics.

[27] Malitson I.H. (1965). Interspecimen Comparison of the Refractive Index of Fused Silica. J. Opt. Soc. Am..

[28] Welsh J.A., Van Der Pol E., Arkesteijn G.J.A., Bremer M., Brisson A., Coumans F., Dignat-George F., Duggan E., Ghiran I., Giebel B. (2020). MIFlowCyt-EV: A framework for standardized reporting of extracellular vesicle flow cytometry experiments. J. Extracell. Vesicles.

[29] Lamichhane T.N., Sokic S., Schardt J.S., Raiker R.S., Lin J.W., Jay S.M. (2015). Emerging roles for extracellular vesicles in tissue engineering and regenerative medicine. Tissue Eng. Part B Rev..

[30] Revenfeld A.L.S., Bæk R., Nielsen M.H., Stensballe A., Varming K., Jørgensen M. (2014). Diagnostic and Prognostic Potential of Extracellular Vesicles in Peripheral Blood. Clin. Ther..

[31] Gudbergsson J.M., Johnsen K.B., Skov M.N., Duroux M. (2016). Systematic review of factors influencing extracellular vesicle yield from cell cultures. Cytotechnology.

[32] Krüger-Genge A., Blocki A., Franke R.-P., Jung F. (2019). Vascular Endothelial Cell Biology: An Update. Int. J. Mol. Sci..

[33] Cines D.B., Pollak E.S., Buck C.A., Loscalzo J., Zimmerman G.A., McEver R.P., Pober J.S., Wick T.M., Konkle B.A., Schwartz B.S. (1998). Endothelial cells in physiology and in the pathophysiology of vascular disorders. Blood.

[34] Letsiou E., Bauer N. (2018). Endothelial Extracellular Vesicles in Pulmonary Function and Disease. Curr. Top. Membr..

[35] Milasan A., Farhat M., Martel C. (2020). Extracellular Vesicles as Potential Prognostic Markers of Lymphatic Dysfunction. Front. Physiol..

[36] Berezin A.E., Berezin A.A. (2020). Extracellular Endothelial Cell-Derived Vesicles: Emerging Role in Cardiac and Vascular Remodeling in Heart Failure. Front. Cardiovasc. Med..

[37] Erdbrügger U., Rudy C.K., Etter M.E., Dryden K.A., Yeager M., Klibanov A.L., Lannigan J. (2014). Imaging flow cytometry elucidates limitations of microparticle analysis by conventional flow cytometry. Cytom. Part A J. Int. Soc. Anal. Cytol..

[38] Hoen E.N.M.N.T., van der Vlist E.J., Aalberts M., Mertens H.C.H., Bosch B.J., Bartelink W., Mastrobattista E., van Gaal E.V.B., Stoorvogel W., Arkesteijn G.J.A. (2012). Quantitative and qualitative flow cytometric analysis of nanosized cell-derived membrane vesicles. Nanomed. Nanotechnol. Biol. Med..

[39] Arraud N., Gounou C., Turpin D., Brisson A.R. (2016). Fluorescence triggering: A general strategy for enumerating and phenotyping extracellular vesicles by flow cytometry. Cytom. Part A J. Int. Soc. Anal. Cytol..

[40] Gardiner C., Ferreira Y.J., Dragovic R.A., Redman C.W.G., Sargent I.L. (2013). Extracellular vesicle sizing and enumeration by nanoparticle tracking analysis. J. Extracell. Vesicles.

[41] Sultanova N., Kasarova S., Nikolov I. (2009). Dispersion Properties of Optical Polymers. Acta Phys. Pol. A.

[42] Brennan K., Martin K., FitzGerald S.P., O’Sullivan J., Wu Y., Blanco A., Richardson C., Mc Gee M.M. (2020). A comparison of methods for the isolation and separation of extracellular vesicles from protein and lipid particles in human serum. Sci. Rep..

[43] Viñals F., Pouysségur J. (1999). Confluence of vascular endothelial cells induces cell cycle exit by inhibiting p42/p44 mitogen-activated protein kinase activity. Mol. Cell. Biol..

[44] Takasugi M., Okada R., Takahashi A., Chen D.V., Watanabe S., Hara E. (2017). Small extracellular vesicles secreted from senescent cells promote cancer cell proliferation through EphA2. Nat. Commun..

[45] Kusakabe A., Okumura N., Hirano H., Kinoshita S., Koizumi N. (2014). The effect of cell density on the adhesion and proliferation properties of cultivated corneal endothelial cells. Investig. Ophthalmol. Vis. Sci..

[46] Heng B.C., Bezerra P.P., Preiser P.R., Law S.K.A., Xia Y., Boey F., Venkatraman S.S. (2011). Effect of cell-seeding density on the proliferation and gene expression profile of human umbilical vein endothelial cells within ex vivo culture. Cytotherapy.

[47] Hamada K., Osaka M., Yoshida M. (2014). Cell Density Impacts Epigenetic Regulation of Cytokine-Induced E-Selectin Gene Expression in Vascular Endothelium. PLoS ONE.

[48] Roederer M., Mays R.W., Murphy R.F. (1989). Effect of confluence on endocytosis by 3T3 fibroblasts: Increased rate of pinocytosis and accumulation of residual bodies. Eur. J. Cell Biol..

[49] Hogg N., Browning J., Howard T., Winterford C., Fitzpatrick D., Gobé G. (1999). Apoptosis in vascular endothelial cells caused by serum deprivation, oxidative stress and transforming growth factor-beta. Endothel. J. Endothel. Cell Res..

[50] Andreu Z., Yáñez-Mó M. (2014). Tetraspanins in extracellular vesicle formation and function. Front. Immunol..

[51] Boyer M.J., Kimura Y., Akiyama T., Baggett A.Y., Preston K.J., Scalia R., Eguchi S., Rizzo V. (2020). Endothelial cell-derived extracellular vesicles alter vascular smooth muscle cell phenotype through high-mobility group box proteins. J. Extracell. Vesicles.

[52] Vagida M.S., Arakelyan A., Lebedeva A.M., Grivel J.-C., Shpektor A.V., Vasilieva E.Y., Margolis L.B. (2016). Analysis of Extracellular Vesicles Using Magnetic Nanoparticles in Blood of Patients with Acute Coronary Syndrome. Biochem. Biokhimiia.

[53] Prattichizzo F., De Nigris V., Sabbatinelli J., Giuliani A., Castaño C., Párrizas M., Crespo I., Grimaldi A., Baranzini N., Spiga R. (2020). CD31 Positive-Extracellular Vesicles from Patients with Type 2 Diabetes Shuttle a miRNA Signature Associated with Cardiovascular Complications. Diabetes.

[54] Lertkiatmongkol P., Liao D., Mei H., Hu Y., Newman P.J. (2016). Endothelial functions of platelet/endothelial cell adhesion molecule-1 (CD31). Curr. Opin. Hematol..

[55] Petzelbauer P., Bender J.R., Wilson J., Pober J.S. (1993). Heterogeneity of dermal microvascular endothelial cell antigen expression and cytokine responsiveness in situ and in cell culture. J. Immunol..

[56] Livshits M.A., Khomyakova E., Evtushenko E.G., Lazarev V.N., Kulemin N.A., Semina S.E., Generozov E.V., Govorun V.M. (2015). Isolation of exosomes by differential centrifugation: Theoretical analysis of a commonly used protocol. Sci. Rep..

